# Neutrophil activity compromises root caries remineralization and alters caries-affected-like dentin

**DOI:** 10.1007/s00784-025-06567-z

**Published:** 2025-10-13

**Authors:** Rinku K Trivedi, Riya H Patel, Asma Wazir, Camila A Zamperini

**Affiliations:** Department of Restorative Dentistry, College of Dentistry, University of Illinois, 801 South Paulina St, Chicago, IL 60612 USA

**Keywords:** Dental caries, Root caries, Neutrophils, Extracellular matrix, Sodium fluoride

## Abstract

**Objectives:**

Root caries (RC) progression involves the dentin extracellular matrix (*d*ECM) degradation by proteolytic enzymes. As neutrophil-derived enzymes have been systemically associated with ECM degradation, we hypothesized that neutrophil activity would: (i) inhibit RC remineralization; and (ii) alter the caries-affected-like dentin.

**Materials and methods:**

In vitro RC were created on human dentin specimens. Half each lesion was covered to keep the initial lesion (IL). The other half (final lesion; FL) was exposed to one of the following groups (*n* = 10): Control (buffer solution; C); Neutrophils (human neutrophils; N); Collagenase (positive control; Col), and then remineralized by pH-cycling and sodium fluoride. RC remineralization was calculated using rhodamine infiltration and cross-sectional microhardness (CSMH). Collagen of the fully demineralized dentin was stained with picrosirius red. The physical properties of the caries-affected-like dentin were investigated by CSMH and loss of mineral density (MD).

**Results:**

Two-way ANOVA and Tukey showed only remineralization in the C group (*p* < 0.001). N did not show any remineralization (*p* = 0.588) between IL and FL. The N and Col groups exhibited changes in collagen integrity and organization. The N and Col groups’ caries-affected-like dentin showed significantly lower CSMH (*p* < 0.001) than the C group, and significant losses (IL-FL) of MD (*p* = 0.012 and *p* = 0.042, respectively). In contrast, no MD loss was observed in the C group (*p* = 0.411).

**Conclusions:**

Neutrophil-derived enzymes compromise RC remineralization and detrimentally affect the physical properties of the caries-affected-like dentin.

**Clinical significance:**

Identifying proteolytic sources involved with *d*ECM degradation during RC progression will pave new research directions.

## Introduction

Root caries (RC) is a common disease among the elderly population. Although there are similarities between RC and crown caries regarding their etiology, there are essential differences in lesion anatomy, location, and tissue structure and composition [1]. During RC progression, the ready exposure of the root dentin extracellular matrix (*d*ECM) to the oral environment introduces a particular dimension to the RC pathophysiology. While it is well recognized that RC lesion progression depends on the degradation of the *d*ECM, the mechanisms underlying this process are not understood. Current evidence supports that the *d*ECM degradation seems to happen by host-derived proteolytic activity, which ultimately leads to lesion progression and compromises remineralization [2–9]. Although this is a well-accepted concept in cariology, there are still significant knowledge gaps regarding the cellular sources of such proteases, including matrix metalloproteinases (MMPs) and cysteine cathepsins, and their exact mechanisms of activation and further interactions.

One of the major producers of proteolytic enzymes is neutrophils, which stand out as indispensable and versatile components of the innate immune system, markedly present in saliva, oral biofilms, and gingival crevicular fluid [10–13]. Despite their well-known protective and antimicrobial functions, these cells play a multifaceted role in various human conditions beyond their role in defense. Neutrophil over-recruitment and activation have been implicated in tissue damage in several systemic diseases [14–20], and, particularly in the oral health context, studies have shown that neutrophils contribute to tissue damage during periodontal disease [21–23].

In the presence of microbial challenges, neutrophils undergo activation to combat bacteria, deploying antimicrobial factors, forming neutrophil extracellular traps (NETs), and generating reactive oxygen species (ROS) [10, 12, 24, 25]. While these mechanisms are critical for defending against microbial threats, the ability to produce proteolytic enzymes leads some researchers to suggest a dual role for neutrophils in the caries context [26], as they may contribute to dentin tissue damage during RC development. It is well known that the remineralization of RC lesions depends on the soundness of the *d*ECM [27]; thus, any damage will substantially interfere with RC remineralization. This study evaluated the remineralization potential of in vitro RC lesions exposed to human neutrophils and the neutrophil-induced physical changes on the caries-affected-like dentin. The tested hypotheses were that (i) human neutrophil-derived products would compromise RC remineralization; (ii) the neutrophil exposure would also compromise the physical properties of the caries-affected-like dentin.

## Materials and methods

### Teeth collection and preparation of root dentin specimens

De-identified human incisor teeth were collected daily on ice from the predoctoral Oral and Maxillofacial Surgery Clinic (IRB#: 2020 − 1557). Subsequently, teeth (*n* = 15) were cleaned by rinsing with water and a scalpel blade and preserved at −20 °C before sectioning. Root dentin specimens measuring approximately 4 × 4 × 2 mm were cut at 2.0 mm below the cementoenamel junction (mesial and distal surfaces; *n* = 30) using a low-speed diamond blade (Isomet 1000, Buehler, Lake Bluff, IL, USA), following a standard method with minor modifications [28, 29]. The specimens were polished under running water with 400-, 600-, 800-, and 1,200-grit silicon carbide (SiC) papers to remove residual cementum and flatten the root surfaces. The dentin specimens were then covered with acid-resistant nail polish (Revlon Corp., New York, NY, USA), except for a central 3 × 3 mm window where experiments were performed. The specimens were then sterilized with ethylene oxide.

### In vitro root caries (RC) formation and experimental groups

In vitro RC formation was induced through a chemical model, by immersing the root specimens in an acidic solution (2.2 mM CaCl_2_.2H_2_O, 2.2 mM KH_2_PO_4_, 50 mM acetate, pH 4.6) at 37 °C for 4 days [27]. Following a thorough rinsing with deionized water, half of each caries lesion was shielded with an acid-resistant nail polish (Revlon Corp., New York, NY, USA) to preserve the baseline lesion (i.e., IL - Initial Lesion) [27]. Then, the other half of each lesion was exposed to one of the three conditions (*n* = 10) for 3 weeks: (1) Control (negative control group; C) - specimens kept in Dulbecco’s Phosphate Buffered Saline (DPBS). (2) Neutrophils (experimental group; N) - specimens exposed to human neutrophils; and (3) Collagenase (positive control group; Col) - specimens kept in collagenase solution.

Human peripheral blood was obtained from a healthy female donor. The blood diluted in DPBS was layered over a double gradient with specific gravities of 1.077 (3 mL) and 1.119 (3 mL) using Histopaque^®^ (MilliporeSigma, Sigma-Aldrich, UK) and centrifuged at 1,820 rpm for 30 min [26]. The resulting neutrophil-rich fraction was carefully extracted, washed twice in DPBS, and resuspended in RBC (red blood cells) lysis buffer (eBioscience, CA, US). Then, the neutrophils were resuspended in calcium-/magnesium-free DPBS at a concentration of 5 × 10^6^ cells/mL [26, 30]. All experiments were conducted within a 1.5-hour timeframe.

For the Col group, bacterial collagenase from *Clostridium histolyticum* (Sigma-Aldrich, St. Louis, MO, USA) was prepared at a 100 µg/mL concentration in DPBS to induce *d*ECM degradation (positive control) [27]. As a negative control group (C), specimens were kept in buffer solution, DPBS. To accommodate the limited neutrophil lifespan and the sustained activity of their degranulation products [26], neutrophils and control solutions were replaced weekly.

### Root caries remineralization

After incubation under different conditions, the specimens were subjected to a daily cycling regimen that commenced and concluded with two-minute fluoride exposure (5000 ppm Sodium Fluoride, NaF; Fisher Chemical, US) to simulate the use of high fluoride concentration toothpaste (twice daily) and optimize remineralization. Between fluoride exposures, six cycles of 30 min of demineralization and 10 min of remineralization were performed [27, 31]. For that, acidic buffer (50 mM acetate; 2.25 mM CaCl_2_·2H_2_O; 1.35 mM KH_2_PO_4_; 130 mM KCl; pH 4.5; 30 min) and neutral buffer (20 mM HEPES; 2.25 mM CaCl_2_·2H_2_O; 1.35 mM KH_2_PO_4_; 130 mM KCl, pH 7.2; 10 min) were freshly prepared and used. All specimens were kept in a neutral buffer overnight. After an 8-day pH-fluoride cycling [31], the specimens were sectioned into two halves, each containing the IL and FL. One half was embedded in epoxy resin for confocal laser scanning microscopy (CLSM), cross-sectional microhardness (CSMH), and 2D and 3D topography imaging by nanoindentation; the mineral density and *d*ECM structure of the other half were analyzed by microcomputed tomography and polarized light microscopy, respectively.

### Caries porosity and depth

Porosity and depth differences between IL and FL within each group (*n* = 10) were investigated by rhodamine infiltration [32, 33] using CLSM (Zeiss LSM 510, Carl Zeiss, Inc., Germany) equipped with an argon laser emitting at a 529 nm excitation wavelength. For that, the specimens were cut perpendicular to the dentin surfaces using a low-speed diamond blade (Isomet 1000, Buehler, Lake Forest, IL, US), embedded in epoxy resin and subjected to polishing on a water-cooled polishing unit (EcoMet 3000, Buehler, Lake Bluff, IL, USA) with abrasive papers (400-, 600-, 800-, and 1,200-grit). Then, the specimens were incubated in a freshly prepared 0.1 mM rhodamine-B solution (Aldrich Chem. Co., Milwaukee, WI, USA) overnight and rinsed for 1 min with deionized water [28, 29].

The depth of rhodamine infiltration indicates the depth of caries lesions (IL and FL), while the fluorescence intensity of the infiltrated rhodamine informs on caries porosity. By calculating the difference in the CLSM values between the initial lesion (IL) and final lesion (FL) stages (i.e., IL – FL), data on the remineralization rate is obtained. For all CLSM analyses, two images (edges of the lesion; IL and FL) were taken for each specimen. The porosity and depth analyses were completed in three standardized locations (central and two equidistant lateral areas) for each image. To achieve this, three cross-sectional linear profiles were traced perpendicularly to the dentin surface, spanning from the top to the bottom of the body of the lesion. The fluorescence intensity (per micrometer) values for each linear profile were then averaged at 10, 20, 30, 40, and 50 micrometers to determine the mean fluorescence intensity at different depths for all the IL and FL. Similarly, the mean depths of caries lesions were determined by averaging the lesion depths in three standardized areas/image. Data on caries porosity and depth were statistically analyzed to assess the assumption of equality of variances and normal distribution of errors using Levene’s and Shapiro-Wilk tests, respectively. Caries porosity and depth were further analyzed by 2-way Analysis of Variance (ANOVA; groups and depth), followed by the Tukey test. A t-test was used to compare IL and FL within each group. All statistical analyses were conducted with a significance level of α = 0.05 using SPSS v20 software.

### 2D and 3D Caries Topography

For the 2D and 3D topography imaging (*n* = 3), specimens embedded and polished in epoxy resin were further polished with 9-, 6-, 3-, and 1-µm diamond suspensions and 0.05 μm alumina suspension (MasterPrep, Buehler, Lake Bluff, IL, USA). The representative Piezo automation scans 2D and 3D (gradient reverse) were performed using a TI980 nanoindenter (Hysitron Bruker, Minneapolis, MN) with a fluid cell Berkovich tip (Frequency: 10 to 100 Hz; Load Force: 800 µN; Load Function: Frequency sweep mode).

### Integrity and organization of the dECM’s collagen

The second half of the specimens (*n* = 3) were fully demineralized in 0.5 M ethylenediaminetetraacetic acid (EDTA; renewed every 2 days) for 21 days at 4 °C. Specimens of root dentin were frozen and cryosectioned (RM 2135, Leica Microsystems GmbH, Wetzlar, Germany) using the optimal cutting temperature (OCT, Scigen Scientific Gardena, CA, USA) as embedding material. Serial 8-µm sections were mounted on glass slides, stained with Picrosirius Red staining following standard methodology [26, 29], and examined by a light and polarized optical microscope (Axioskop 40, Carl Zeiss Microscopy, Thornwood, NY, USA).

### Cross-sectional microhardness (CSMH)

The effect of the different experimental conditions on the final CSMH (*n* = 5) within the most superficial 200 μm, encompassing the body of the RC lesions and the caries-affected-like dentin, was evaluated using a microhardness tester (LM 700 AT, Leco, MI, US). The measurements were performed with a Knoop tip (KHN) [31, 34] using a 25 g load force for 10 s in 3 locations within the FL for each specimen. Ten in-depth measurements were completed every 20 μm in different depths from the surface (20 to 200 μm) in three locations located approximately 300 μm away. The three measurements made at three locations were averaged for each depth. Then, CSMH data were statistically analyzed to determine the assumption of equality of variances and normal distribution of errors using Levene’s and Shapiro-Wilk tests, followed by 2-way ANOVA (groups and depth) and Scheffe tests.

### Mineral density (MD) of the Caries-Affected dentin

Microcomputed tomography (*n* = 6) was used to assess the differences (IL-FL) in MD of the caries-affected-like dentin after exposure to different experimental conditions [27, 28]. The second half of the specimens were fixed in 10% neutral buffered formalin for 24 h, placed securely in a specimen holder, and kept soaked with phosphate-buffered saline during scanning. The scanning parameters used were 55 kVp energy (X-ray voltage), 109 µA intensity, and 500 ms integration time using 3.4 μm voxels/resolution, with 2,048 × 2,048 reconstructed images (µCT-50; Scanco Medical, Wayne, PA, USA). The MD of the caries-affected-like dentin was estimated using a standard calibration phantom provided by the manufacturer. The MD was calculated in two areas (below IL and FL)/specimen using a standardized volume of interest. Data presented as loss of MD (IL-FL) were statistically analyzed following the determination of the assumption of equality of variances and normal distribution of errors using Levene’s and Shapiro-Wilk tests. Differences in MD among groups for IL and FL were analyzed by Analysis of Variance (ANOVA), and a t-test was used to compare the loss of MD within groups (IL-FL).

## Results

### RC porosity, depth, and topography

**Fig. 1:**
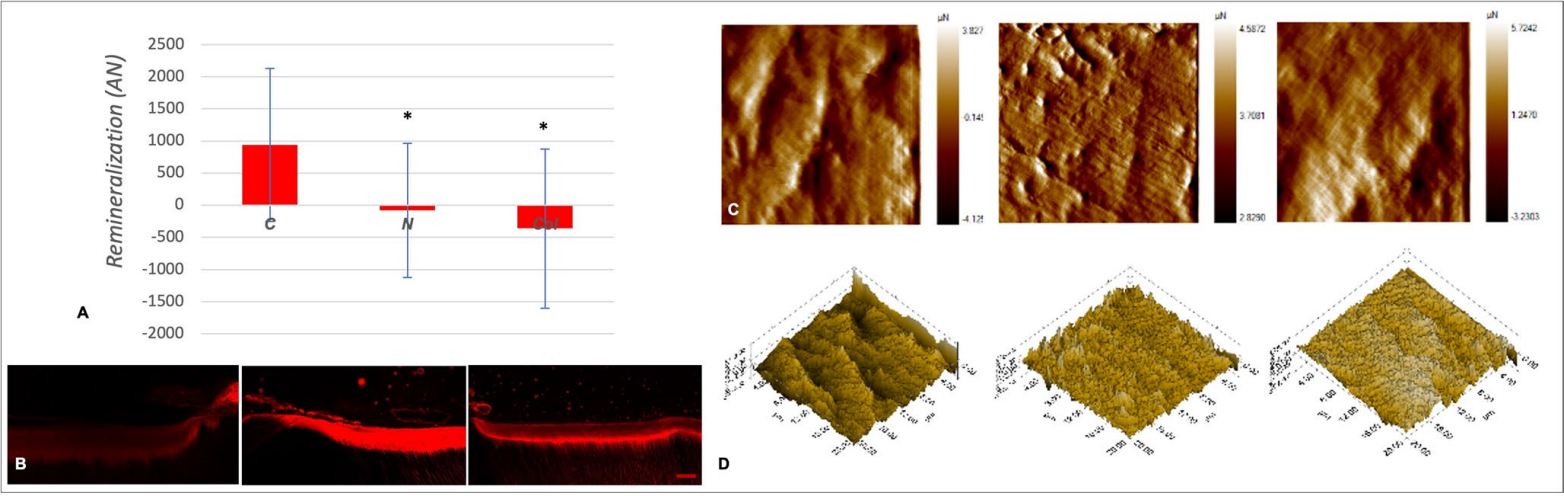
Means and standard deviations (**A**) of RC remineralization achieved after dECM being exposed to control (**C**), neutrophils (N) and collagenase (Col) conditions. Two-way ANOVA followed by Tukey test show significant differences between N (p < 0.001) and Col (p < 0.001) groups compared to C group (*). Representative images of the rhodamine infiltration (**B**) highlighting the remaining final RC lesions (FL) for all groups; Scale bar: 100 µm. Fig. C and D show differences in the 2D and 3D surface topography, respectively, as imaged by triboindenter. Image sequence: C, N and Col groups for all analyses (**A**-**D**).

For IL and FL porosity, two-way ANOVA revealed a significant effect of experimental groups (*p* < 0.001), no effect of depth (*p* = 0.989), and no interaction between factors (*p* = 0.995). Tukey test showed statistically significant differences in remineralization between N (*p* < 0.001) and Col (*p* < 0.001) groups compared to the C group (Fig. 1A and B). The remineralization reached in the C group was significantly higher than that observed in the N and Col groups, which did not differ from each other (*p* = 0.478). The t-tests within each group (Fig. 1B) detected significant remineralization (FL-IL) for the C group (*p* < 0.001), and a statistically significant increase in porosity (demineralization) for the Col (*p* = 0.045) group. In contrast, no statistically significant change in remineralization between IL and FL was noted within the N group (*p* = 0.588).

There was a slight increase in caries depth after cycling, as indicated by the t-test (*p* ≤ 0.01), regardless of experimental groups, with no statistically significant difference among them (*p* = 0.260). The increases in mean RC depth (FL-IL) detected for the C, N, and Col groups were, respectively: 14 μm (standard deviation (SD) of 15 μm), 28 μm (SD: 23 μm), and 25 μm (SD: 19 μm).

The topography imaging also shows remineralization impairments (Fig. 1C and D). The 2D image of the C group reveals a standard dentin structure where the dentinal tubules and the intertubular dentin can be identified; conversely, no clear differentiation between dentinal tubules and intertubular dentin can be visualized for the RC lesions exposed to N and Col. The 3D topography confirms the differences among groups: peaks of remineralization can be identified for the C group, while flatter surfaces are observed for the enzymatically degraded groups.

### Integrity and organization of the dECM’s collagen

**Fig. 2:**
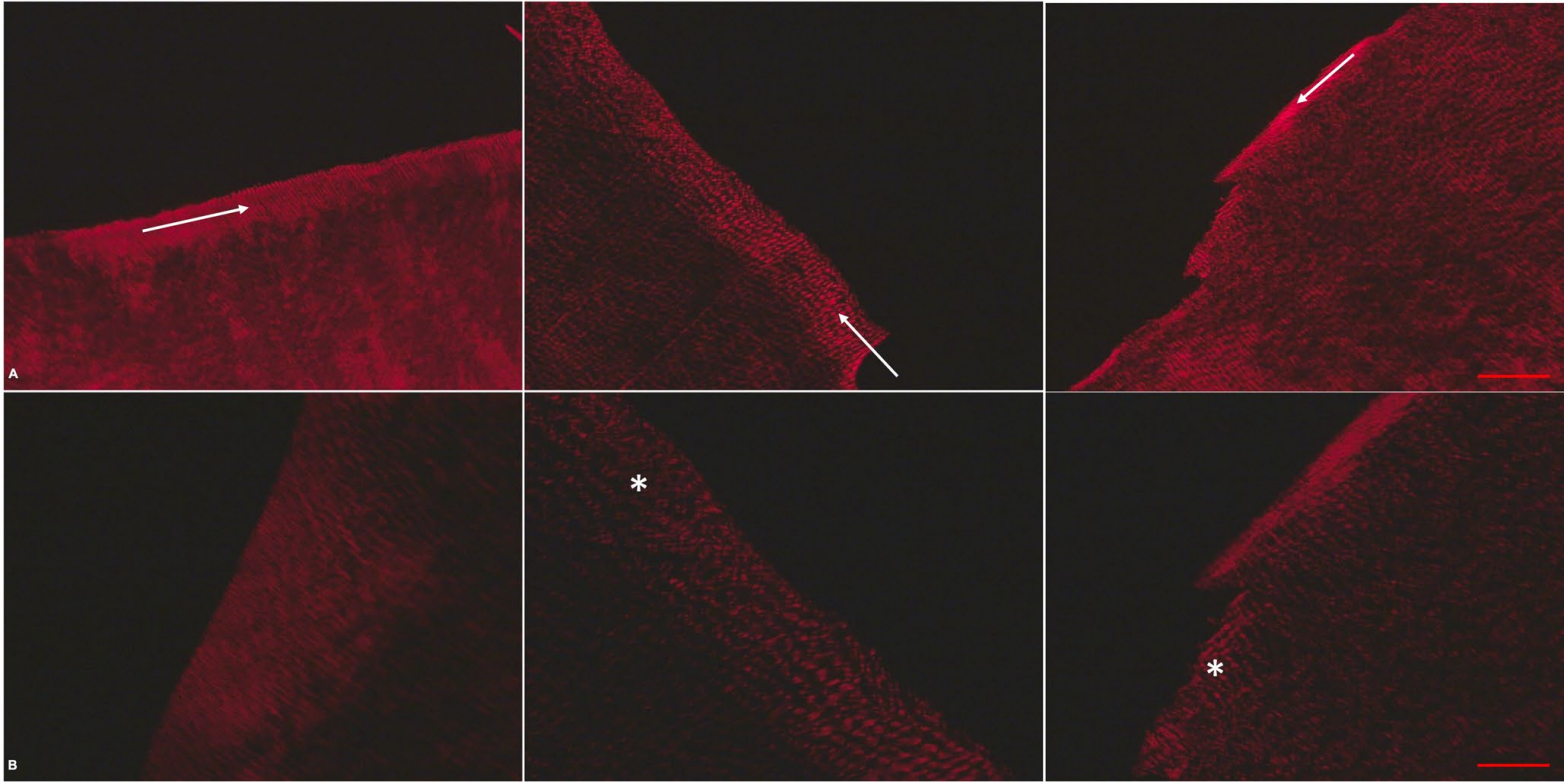
Figures A and B: histological images under polarized light microscope (20× and 40×, respectively) showing differences in collagen packing and organization. Some degree of collagen crosslinking is observed for all groups (white arrows in Fig. A); a less dense collagen network and more disorganized collagen fibers were observed in the N and Col groups (white asterisk in Fig. B; 40×), with an advanced degree of tissue degradation in the Col group. A denser and well-packed collagen network with more organized collagen fibers was observed for the C group. Regardless of the groups, specimens present a mature collagen network (red collagen fibers under a polarized light microscope). Image sequence: C, N, and Col groups (A and B). Scale bar: 100 μm (**A**) and 50 μm (**B**)

Qualitative histological analyses under a polarized light microscope indicate noticeable differences among groups. Polarized light microscope (Fig. 2A and B) shows differences in collagenous structure, packing, and organization. Although the three groups presented some degree of collagen crosslinking in the RC area (white arrows in Fig. 2A), a less structured and less dense collagen network and more disorganized collagen fibers were observed in the N and Col groups (white asterisk in Fig. 2B), with an advanced degree of tissue degradation in the Col group. On the other hand, a denser and very homogeneous collagen network with structured collagen fibers was observed for the C group. Regardless of the groups, specimens present a mature collagen network (red collagen fibers under a polarized light microscope).

### CSMH and MD of the caries-affected-like dentin

**Fig. 3:**
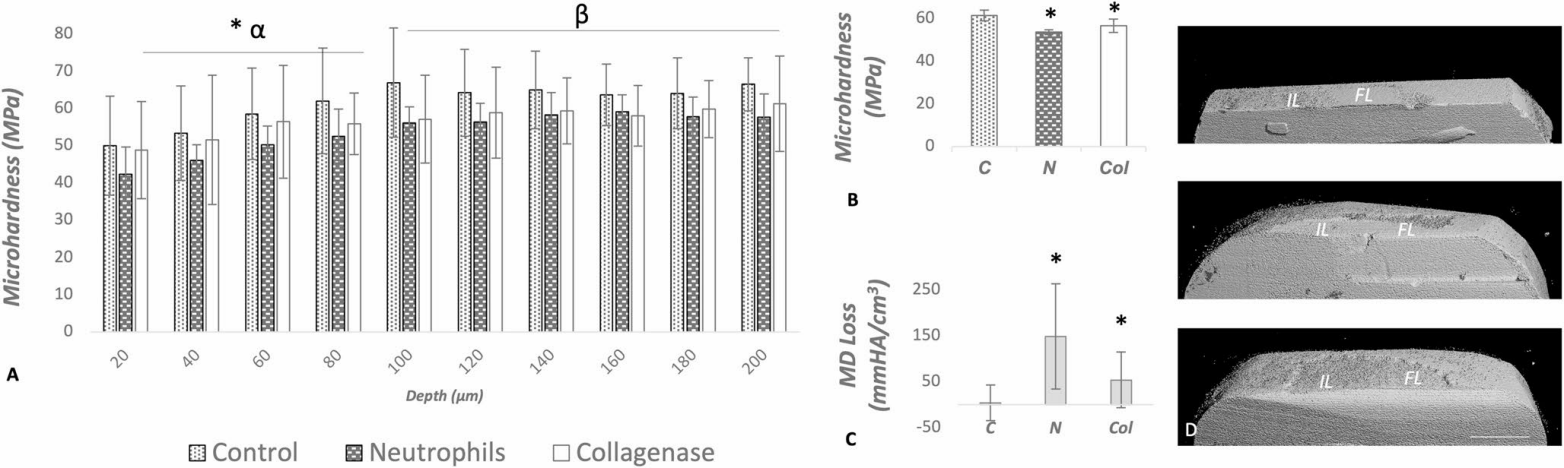
CSMH of in vitro remineralized RC lesions (α) and caries-affected (β) dentin showing consistently lower microhardness in the N and Col groups in different depths (**A**). Lower microhardness means in the N and Col groups, regardless of depth (**B**). Fig. C shows significantly greater mineral density (MD) loss in the caries-affected dentin in the N and Col groups. 3D representative images of the IL and FL for the C, N, and Col groups (**D**). Each bar represents the Mean and ± SD. C: Control; N: Neutrophils; and Col: Collagenase. * Statistically significant differences among groups (**A**) compared to Control (**B** and **C**). Scale bar: 1 mm

For CSMH (Fig. 3A and B), Two-way ANOVA showed effects of both factors (groups and depth; *p* < 0.001) with no interaction among them (*p* = 0.999). Scheffe’s test pointed to significant differences (*p* < 0.001) in mean microhardness of the N (53.65 MPa) and Col (56.70 MPa) groups compared to the Control group (61.43 MPa), regardless of depth. Lower microhardness was observed within the most superficial 80 μm, irrespective of the experimental groups.

There were no statistically significant differences among groups for MD of the caries-affected-like dentin below the IL (*p* = 0.254) and FL (*p* = 0.193). However, when analyzing loss of MD of the caries-affected-like dentin within each group (IL-FL), there were statistically significant differences in the N (*p* = 0.012) and Col (*p* = 0.042) groups (Fig. 3C and D). In contrast, no significant change was detected for the C group (*p* = 0.411).

## Discussion

This study focused on investigating the impact of the human neutrophil activity on the in vitro RC remineralization and on the physical properties of the caries-affected-like root dentin. Unlike other studies [35, 36], this investigation shows how neutrophil activity may interfere with caries development and management. The results of caries porosity show a marked remineralization impairment for specimens exposed to human neutrophils. While the fluoride-containing cycling could partially remineralize the specimens in the Control group, the ones exposed to neutrophils and collagenase exhibited comparable lack of remineralization or demineralization, respectively. The decreased microhardness of the final RC lesions in the N and Col groups further supports these findings. There is an expressive body of evidence associating the breakdown of the *d*ECM during caries progression with dentin and salivary proteolytic enzymes, including metalloproteinase-8 (MMP-8; collagenase-2) and MMP-9 (a 92-kDa gelatinase) [2–9]. These studies suggest that host-derived collagenases initially degrade collagen molecules during microbial acid-induced dentin demineralization. Then, the denatured collagen is further degraded by additional proteases, such as gelatinases and peptidases. Human neutrophils are immune cells rich in proteases, including MMP-8, MMP-9, neutrophil elastase, proteinase 3, and cathepsins, released during the neutrophil degranulation, with outstanding potential to degrade *d*ECM. In turn, a damaged *d*ECM by neutrophil activity cannot support a successful remineralization, as observed in our findings. Similarly, collagenase from *Clostridium histolyticum* is known for its ability to degrade collagen chains (Toledano et al., 2007), and it was purposely added in this investigation to degrade *d*ECM and, thus, compromise the remineralization (positive control). The topography analyses confirmed the same patterns of remineralization observed for all three groups by the confocal microscopy and microhardness data. The 2D and 3D topography images show that the N and Col groups presented a structurally damaged dentin, while the C group remained intact structurally and supported, to some extent, RC remineralization. A lower degree of collagen packing and organization was observed when specimens were exposed to neutrophils and collagenase, suggesting some *d*ECM degradation in both groups. Thus, based on the remineralization, lesion topography, and histological analyses, our first tested hypothesis was accepted.

In addition to the pivotal role of the *d*ECM as a remineralization network, it has been observed that an intact or reinforced *d*ECM is also essential to inhibit dentin lesion progression [37]. The increased caries porosity (demineralization) observed within the Collagenase group illustrates the protective effect of the dECM against lesion progression. This has been related to the ampholytic nature of its proteins that may play a buffering role in neutralizing caries-inducing acids [38]. Thus, it would be reasonable to expect deeper FL in the groups with potentially degraded *d*ECM. Although there were numerical increases in RC depth for the N and Col groups, these differences did not reach statistical significance. This might be partially attributed to the fluoride’s protective effect against caries progression. In our fluoride-containing pH-proteolytic cycling model, 5000 ppm sodium fluoride (NaF, 2×daily) was added to the 8-day cycling to induce RC remineralization. It is well known that fluoride has multiple effects as an anticaries agent [28, 39–43], including the impact against demineralization. Thus, it seems logical to assume that the high fluoride concentration, to some extent, avoided caries progression for all groups and prevented the development of significantly deeper RC lesions in the neutrophils and collagenase conditions.

Still in alignment with the importance of the *d*ECM in slowing down lesion progression, another focus of our study was to investigate the neutrophil degradative effect in the deeper dentin (below the body of the lesion; herein named as caries-affected-like dentin), assuming that the caries-affected-like dentin located below lesions with potentially degraded *d*ECM would be significantly more affected. Based on the CSMH data, we found that the lesions exposed to neutrophils and collagenase presented lower microhardness up to 200 μm in depth, at least, revealing neutrophil degradative effect not only in the body of the lesion (~ 80 μm) but also below the lesion. This observation is also supported by the MD data that showed loss of mineral density (IL-FL) of the caries-affected-like dentin in the specimens exposed to the enzymatic challenges. In contrast, no MD loss was detected in the C group. Thus, the second tested hypothesis was also accepted. These enzymatically induced changes in the physical properties of the caries-affected-like dentin can be explained by dentin permeability, mainly represented by the entrance of dentinal tubules that may function as channels for diffusion of pH cycling solutions and enzymes. Thus, the in vitro RC model herein proposed seems to have provided us with a second opportunity, where our hypotheses could be confirmed: for the C group, the superficially demineralized bottom of the lesion and/or entrance of the dentinal tubules are efficiently remineralized by the fluoride-containing cyclings due to the presence of a sufficiently intact *d*ECM. This successful demin-remineralization process protects the dentin under the body of the lesion from further physical changes; conversely, the enzymatically-induced *d*ECM degradation in the N and Col groups prevented the full remineralization in the bottom of the lesion and/or entrance of the dentinal tubules, making the underneath dentin, the caries-affected-like dentin, more porous and, consequently, compromising its microhardness and mineral density.

In this study, we used an in vitro pH-cycling model designed to replicate the fluctuating mineral saturation and pH levels associated with a cariogenic diet, while maintaining the soundness of the *d*ECM to allow some degree of remineralization (C group). Our positive control group incorporated a bacterial enzymatic challenge to emulate the dECM breakdown by host-derived enzymes. Adding the 5000 ppm NaF simulated an evidence-based non-restorative approach to control RC [44]. We tried to optimize the fluoride’s remineralizing property by associating repetitive demineralization-remineralization episodes with high fluoride concentration. This complex in vitro model aims to consider the interplay of clinically relevant factors (including fluoride and the tissue’s organic and inorganic components) in dentin remineralization. According to previous studies [45, 46], the natural caries-affected dentine typically falls within the 25–40 KHN range. The relatively higher cross-sectional microhardness values observed in our study for caries-affected-like dentin are not unexpected, given the inherent limitations of the in vitro model and the fluoride-containing remineralization protocol employed. In recognition of this, the term “caries-affected-like dentin” has been chosen to reflect the nature of our experimental conditions more accurately. Despite these limitations, the model remains relevant as it clearly demonstrates remineralization dynamics—or the lack thereof. The maintenance of an altered dentin (up to at least 200 micrometers) in the groups under proteolytic challenges highlights the appropriateness of this cycling as a relevant in vitro caries model that aims to account for the role of the *d*ECM [45, 46].

We acknowledge the lack of microbial biofilms and their ability to modulate the neutrophil role in the RC context as one limitation of the present study, and thus, will be considered in the future. Despite that, we still believe the fluoride-containing pH-proteolytic cycling model proposed herein as a critical mechanistic approach to understand the *d*ECM-neutrophil interactions during RC progression.

The clinical relevance of the present study lies not in the direct translation of the remineralization outcomes but rather in the innovative conceptual framework, which considers neutrophils as an active contributor to the complex interplay among dental biofilms, dental tissues, and diet in the context of caries management. Further investigations are warranted to comprehensively understand the mechanisms implicated in the *d*ECM degradation and caries progression, particularly those involving interactions among caries-related biofilms, neutrophil enzymes, and dental tissues.

## Conclusion

Based on the findings of this study, we can conclude that human neutrophil activity compromises RC remineralization and induces more profound physical changes within caries-affected root dentin.

## Data Availability

All data generated or analyzed during this study are included in this manuscript. Further enquiries can be directed to the corresponding author.

## Declarations

This study protocol was reviewed and approved by the University of Illinois Chicago (UIC) Institutional Review Board (IRB, approval number 2020 − 1557).
